# Neoadjuvant Apalutamide With or Without Abiraterone Acetate Plus Prednisone and Gonadotropin-Releasing Hormone Agonist Prior to Nerve-Sparing Radical Prostatectomy in High-Risk Prostate Cancer: Protocol for a Randomized Phase 2 Trial

**DOI:** 10.2196/97886

**Published:** 2026-11-03

**Authors:** Anthony Y Zhang, Mark N Stein, Saum Ghodoussipour, Isaac Y Kim

**Affiliations:** 1 Department of Urology, School of Medicine, Yale University, 333 Cedar Street, New Haven, CT, 06510, United States, 1 877-925-3637; 2 Division of Medical Oncology, College of Physicians and Surgeons, Columbia University, New York, NY, United States; 3 Division of Urology, Department of Surgery, Robert Wood Johnson Medical School, New Brunswick, NJ, United States

**Keywords:** prostatic neoplasms, neoadjuvant therapy, apalutamide, abiraterone acetate, prostatectomy, erectile dysfunction, urinary incontinence, randomized controlled trial

## Abstract

**Background:**

Radical prostatectomy (RP) is an accepted, definitive treatment for appropriately selected men with high-risk localized prostate cancer (PCa). However, neoadjuvant androgen receptor inhibition prior to RP is not currently the standard of care. Wide resection of the neurovascular bundles may improve local oncologic control but can adversely affect postoperative erectile function and urinary continence. Apalutamide directly inhibits the androgen receptor, while abiraterone acetate inhibits cytochrome P450 17A1–mediated androgen synthesis and is often administered with prednisone. Gonadotropin-releasing hormone (GnRH) agonists further suppress testicular androgen production. Intensified androgen pathway inhibition prior to surgery may therefore downstage localized disease and facilitate bilateral nerve-sparing RP.

**Objective:**

This study was defined to evaluate whether 3 months of neoadjuvant apalutamide-based therapy before RP improves postoperative potency recovery by 12 months compared with immediate RP in men with high-risk localized PCa. Secondary objectives include evaluation of postoperative continence, pathological outcomes, biochemical recurrence, perioperative outcomes, and quality of life (QoL).

**Methods:**

This is a prospective, multicenter, randomized phase 2 clinical trial (ClinicalTrials.gov: NCT02949284). Eligible patients with high-risk PCa were randomized 1:1:1 to (1) apalutamide 240 mg daily for 3 months followed by RP, (2) apalutamide 240 mg daily with abiraterone acetate 1000 mg plus prednisone 10 mg daily and a GnRH agonist for 3 months followed by RP, or (3) immediate RP without neoadjuvant therapy. The primary end point is potency recovery by 12 months postoperatively, defined as the ability to penetrate in sexual intercourse in more than half of attempts. Secondary end points include continence recovery, pathological response, positive surgical margins, biochemical recurrence, perioperative outcomes, and QoL. The primary comparisons evaluate each experimental arm against the surgery-only arm. Pairwise comparisons of the primary end point will use 1-sided Fisher exact tests with a Bonferroni adjustment. Additional continuous and categorical outcomes will be analyzed using appropriate nonparametric or categorical tests, and biochemical recurrence-free survival will be assessed using Kaplan-Meier analysis.

**Results:**

As of March 2026, patient enrollment has been completed. A total of 96 patients provided consent and were screened, 88 were randomized, and 73 received the assigned treatment and were included in the final analysis cohort. Study accrual began on June 20, 2017, and enrollment was completed in June 2024. The prespecified postoperative follow-up period extended beyond 24 months, with a study data cutoff date of June 30, 2025. Data collection for the primary and secondary outcomes has been completed, with final expected results anticipated in January 2027.

**Conclusions:**

This randomized phase 2 trial was designed to assess whether short-term androgen pathway inhibition can improve postoperative functional outcomes while maintaining oncologic outcomes in men undergoing nerve-sparing RP for high-risk localized PCa.

## Introduction

In men with high-risk localized prostate cancer (PCa) who opt for surgery, wide resection of the neurovascular bundles (NVBs) ensures the optimal oncologic control. However, the cost of such aggressive local tumor control is poor quality of life (QoL), including profound erectile dysfunction and incontinence [1,2]. Thus, downstaging local disease prior to radical prostatectomy (RP) in men with high-risk localized PCa may improve postsurgery QoL by rendering nerve-sparing RP more feasible.

Neoadjuvant androgen deprivation therapy (ADT) before RP has been investigated for decades in men with high-risk PCa. Earlier studies demonstrated reductions in positive surgical margin rates but did not demonstrate improvements in oncologic outcomes [3,4]. Similarly, subsequent evaluations of neoadjuvant chemohormonal therapy prior to RP did not demonstrate significant improvements in biochemical progression-free survival. Nevertheless, the consistent reduction in adverse pathological features following preoperative androgen suppression provides a biological rationale for evaluating whether tumor downstaging may also facilitate a more conservative surgical strategy.

Recently, intensified androgen receptor pathway inhibition has expanded the treatment landscape for advanced PCa. Apalutamide is a next-generation nonsteroidal androgen receptor pathway inhibitor that directly inhibits androgen receptor signaling [5]. Apalutamide has shown a favorable oncologic profile compared with first-generation antiandrogens and entered clinical evaluation across multiple PCa disease states, ultimately receiving the Food and Drug Administration approval for patients with nonmetastatic castration-resistant PCa and metastatic castration-sensitive PCa [6,7].

Abiraterone acetate inhibits cytochrome P450 17A1–mediated androgen biosynthesis and, when administered with prednisone and ADT, has demonstrated improved survival in metastatic PCa. Gonadotropin-releasing hormone (GnRH) agonist therapy further suppresses testicular androgen production. Thus, combining direct androgen receptor blockade with inhibition of androgen synthesis and gonadal androgen suppression provides a biologically plausible strategy for achieving more complete androgen pathway inhibition [8-10].

Since the conception of the present study, further clinical investigation has supported the relevance of intensified perioperative androgen pathway inhibition in high-risk localized PCa. The phase 3 Perioperative Treatment with Erleada United with Surgery trial evaluated apalutamide combined with ADT administered both before and after RP [11]. Although the perioperative sandwich treatment strategy differs substantially from the short-course neoadjuvant-only approach evaluated in the present protocol, these findings further support investigation of androgen pathway inhibition in the surgical management of high-risk localized disease.

In this context, we hypothesized that apalutamide-based neoadjuvant monotherapy or combination therapy prior to RP in men with high-risk PCa may improve postoperative potency recovery by downstaging local disease and rendering bilateral nerve-sparing RP more feasible. We therefore designed a prospective, randomized, 3-arm phase 2 trial to evaluate the effect of neoadjuvant apalutamide alone or in combination with abiraterone acetate plus prednisone and a GnRH agonist before RP in men with high-risk localized PCa. The primary end point was postoperative potency by 12 months, and secondary end points included postoperative continence, pathological outcomes, biochemical recurrence, and postsurgical QoL.

## Methods

### Study Design

This study is a prospective, multicenter, open-label, randomized phase 2 study investigating the effect of neoadjuvant apalutamide or apalutamide with abiraterone acetate plus prednisone and a GnRH agonist (Lupron [AbbVie] or Zoladex [AstraZeneca]) on post-RP potency and continence in men with high-risk localized PCa. We propose that the use of apalutamide alone or apalutamide with abiraterone acetate plus prednisone and a GnRH agonist for 3 months preoperatively will downstage the local tumor such that bilateral NVB sparing becomes more feasible, resulting in the improvement of postsurgical potency without compromising oncologic outcomes. The protocol-specified study duration was 5 years, including 2 years of accrual, 2 years of follow-up, and 1 year of analysis.

This protocol was developed at and sponsored by the Rutgers Cancer Institute of New Jersey (CINJ) and received funding and medication support from Janssen Scientific Affairs. The protocol version date was April 13, 2021 (CINJ #081603).

### Ethical Considerations

This study was approved by the institutional review boards of Rutgers (Pro20160000563) and Yale (Human Investigation Committee number: 2000031291), and all patients provided written informed consent prior to enrollment. The protocol specified that informed consent must be obtained before any study-specific procedures. Additional safeguards were included for non–English-speaking participants through interpreter-assisted consent procedures and institutional review board–approved short-form documentation, when applicable.

### Study Population

Eligible participants were men with histologically confirmed prostate adenocarcinoma and high-risk localized disease. High-risk disease was defined as a Gleason score of 8 or higher or a prostate-specific antigen (PSA) level greater than or equal to 20 ng/mL with 2 or more positive cores. Patients were required to have clinical stage T3 or less; no evidence of metastasis; an Eastern Cooperative Oncology Group (ECOG) performance status of 0 or 1; and adequate baseline laboratory values, including hemoglobin, platelet count, albumin, renal function, potassium, bilirubin, and hepatic transaminases within protocol-defined limits. Clinical stage T3 or less remained eligible for the protocol-directed nerve-sparing approach; palpable disease and magnetic resonance imaging (MRI)–detectable lesion location were not specified as absolute contraindications to nerve sparing.

Key exclusion criteria included clinical stage T4 disease, metastatic PCa, a seizure history or conditions that predisposed patients to seizures, a recent major cardiovascular or thromboembolic event, clinically significant ventricular arrhythmias, moderate or severe hepatic impairment, and warfarin use. Given the known association between androgen receptor antagonists and seizure threshold reduction, patients taking medications known to lower the seizure threshold were required to discontinue or substitute those agents prior to study entry.

### Recruitment and Enrollment

Participants were recruited from urologic and medical oncology clinics at CINJ (New Brunswick, New Jersey) and Yale Cancer Center (New Haven, Connecticut). Centralized enrollment procedures used the OnCore clinical trials management system. Following signed consent, patients were registered, screened using a study-specific eligibility checklist, and randomized only after confirmation of protocol eligibility.

### Randomization and Interventions

Patients were randomized 1:1:1 to 1 of 3 treatment arms, with the full schema presented in Figure 1. Surgery consisted of RP with extended pelvic lymph node dissection (ePLND). Prior to surgery, study participants randomized to arm 1 received apalutamide (240 mg; 4×60 mg tablets) orally once daily for 3 months. Patients randomized to arm 2 received apalutamide (240 mg; 4×60 mg tablets), abiraterone acetate (1000 mg; 4×250 mg tablets), and prednisone (10 mg; 2×5 mg tablets to be taken in the morning) orally once daily without food for 3 months. Patients in arm 2 also received a subcutaneous injection of a GnRH agonist (Lupron, 22.5 mg intramuscularly, or Zoladex, 10.8 mg subcutaneously). Patients in arm 2 took apalutamide 1 week (±2 days) before receiving the GnRH agonist and abiraterone acetate with prednisone (AAP). Both apalutamide and abiraterone acetate were securely stored in the pharmacy in charge of dispensing the investigational products. Surgery was performed as early as possible but not earlier than 2 weeks after completion of apalutamide treatment in the treatment arms (arms 1 and 2).

The selected drug doses reflected established clinical dosing and available safety and pharmacokinetic data at the time of protocol development. Apalutamide 240 mg once daily corresponded to the established clinical dose subsequently used in pivotal phase 3 studies, including the Targeted Investigational Treatment Analysis of Novel Anti-Androgen study [6]. The combination of apalutamide with abiraterone acetate 1000 mg and prednisone 10 mg daily was selected in consultation with the study sponsor and was supported by pharmacokinetic data evaluating concurrent apalutamide and abiraterone acetate plus prednisone [8]. The GnRH agonist regimens, leuprolide 22.5 mg intramuscularly or goserelin 10.8 mg subcutaneously, represented standard 3-month depot formulations. The 3-month neoadjuvant duration was selected to allow sufficient androgen pathway suppression and potential local tumor downstaging while minimizing delays to definitive RP and, based on the principal investigator’s surgical experience, limiting the potential for periprostatic fibrosis associated with more prolonged preoperative systemic therapy.

Patients randomized to arm 3 immediately underwent surgery without receiving neoadjuvant treatment.

**Figure 1.**
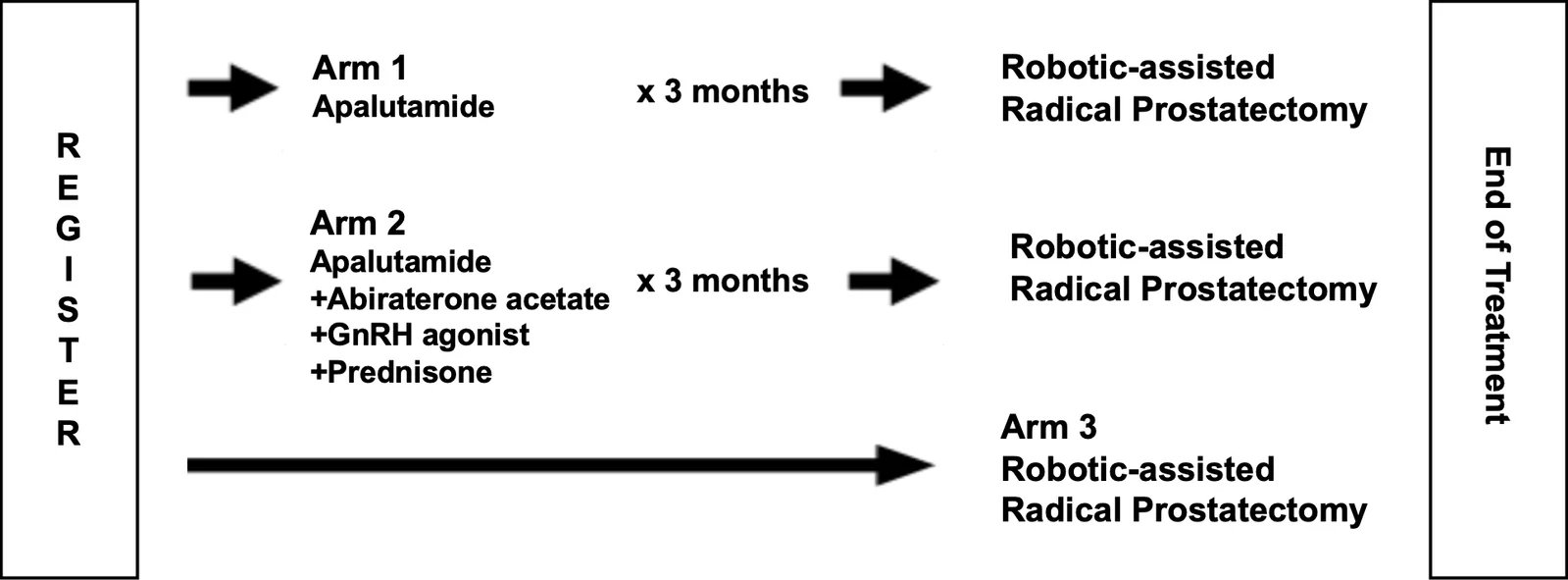
Study schema of a randomized phase 2 trial evaluating neoadjuvant androgen receptor–targeted therapy prior to radical prostatectomy (RP) in men with high-risk prostate cancer. Eligible patients were enrolled and randomized 1:1:1 to (1) apalutamide monotherapy for 3 months, (2) apalutamide with abiraterone acetate plus prednisone and gonadotropin-releasing hormone (GnRH) agonist for 3 months, or (3) immediate robot-assisted RP. The primary end point was recovery of potency by 12 months after RP.

### Surgical Management

Patients deemed surgically eligible underwent robot-assisted RP with ePLND. Bilateral NVB sparing was the planned surgical approach and was performed in all patients undergoing RP; unilateral or wide NVB resection was not performed. Pathological assessment after surgery included margin status, pathological stage, and nodal status, with central genitourinary pathology review within each institution’s department.

Perioperative outcomes included major and minor complications based on the Clavien-Dindo classification system, estimated blood loss, operative time, and length of hospital stay.

### Study Assessments

#### Baseline Assessments

All participants underwent pretreatment evaluation within 30 days before day 1, unless otherwise specified. Baseline assessments included medical history; clinical stage; biopsy Gleason score; demographics; physical examination; vital signs; electrocardiogram; concomitant medications; complete blood count; serum chemistry; PSA; testosterone and androgen panel; urinalysis; bone scan; computed tomography or MRI of the abdomen or pelvis as indicated; multiparametric prostate MRI; ECOG performance status; and patient-reported outcome measures, including the American Urological Association (AUA) Symptom Score, Sexual Health Inventory for Men (SHIM), and Expanded Prostate Cancer Index Composite (EPIC).

#### On-Treatment Assessments

For men randomized to arms 1 and 2, protocol treatment evaluations were performed at day 1 and at weeks 5, 9, and 13, with surgery planned during weeks 14 to 17. Serial assessments included physical examination; vital signs; laboratory testing, including PSA, serum testosterone and androgen levels, urinalysis, and thyroid-stimulating hormone; adverse event monitoring; and repeat multiparametric prostate MRI before surgery. Patient-reported outcomes were collected at each visit during treatment.

### Postoperative Follow-Up

Each patient underwent postoperative follow-up through 24 months. The follow-up schedule included visits at week 1; months 1, 2, and 3; and months 6, 9, 12, 15, 18, 21, and 24. Follow-up assessments included physical examination; adverse event documentation; PSA; selected laboratory studies, including serum testosterone and androgen levels and urinalysis; ECOG performance status; and QoL assessments using the AUA Symptom Score, SHIM, and EPIC models (Table 1).

**Table 1.** Assessments throughout the clinical trial^a^.

Assessment	Screening or baseline	Neoadjuvant period (weeks 1‐13)^b^	Before surgery	Postoperative follow-up^c^
Eligibility and enrollment				
Informed consent	✓			
Medical history and demographics	✓			
Clinical staging (PSA^d^, biopsy Gleason, and imaging)	✓			
Clinical assessments				
Physical examination and vital signs	✓	✓	✓	✓
ECOG^e^ performance status	✓	✓		✓
Concomitant medications	✓	✓	✓	✓
Adverse event monitoring		✓	✓	✓
Laboratory assessments				
Complete blood count	✓	✓		✓
Serum chemistry	✓	✓		✓
Prostate-specific antigen	✓	✓	✓	✓
Testosterone and androgen panel	✓	✓		✓
Thyroid-stimulating hormone	✓	X		
Urinalysis	✓	✓		✓
Imaging				
Multiparametric prostate MRI^f^	✓		✓^g^	
Patient-reported outcomes				
AUA Symptom Score	✓	✓		✓
SHIM^h^ (sexual function)	✓	✓		✓
EPIC^i^ (quality of life)	✓	✓		✓
Biospecimen collection				
Blood and urine collection for biobanking	✓	✓		
Prostate tissue (surgical specimen)				✓
Surgical and pathological outcomes				
Radical prostatectomy				✓
Pathological staging and margin status				✓
Perioperative complications				✓
Follow-up outcomes				
Continence assessment				✓
Erectile function assessment				✓
Biochemical recurrence (PSA-based)				✓

a Summary of the schedule of outcome assessments across all study arms. Arms 1 and 2 underwent serial assessments during the 3-month treatment period and repeat evaluation before radical prostatectomy. All patients underwent standardized postoperative follow-up.

b Neoadjuvant period applies to arm 1 and arm 2 only.

c Postoperative follow-up includes all visits after surgery.

d PSA: prostate-specific antigen.

e ECOG: Eastern Cooperative Oncology Group.

f MRI: magnetic resonance imaging.

g Repeat magnetic resonance imaging performed after completion of neoadjuvant therapy in treatment arms.

h SHIM: Sexual Health Inventory for Men.

i EPIC: Expanded Prostate Cancer Index Composite.

### Outcomes

The primary end point was potency rate by 12 months after RP. Secondary end points included complete pathological response rate (pathologic stage T0), positive surgical margin rate, biochemical recurrence rate, postoperative continence rate, change in tumor volume on pelvic MRI after neoadjuvant therapy, and postsurgical QoL.

Continence was defined using EPIC question 5 as the use of no pads, and potency was defined based on EPIC question 19 and SHIM questions 2 and 4 as the ability to achieve penetration and complete sexual intercourse in more than half of attempts.

Biochemical recurrence was defined using PCa Clinical Trials Working Group 2 criteria as a rising PSA level greater than 0.2 ng/mL, confirmed by a second value at least 3 weeks later.

### Biospecimen and Correlative Studies

Biospecimens were prospectively collected and banked to permit future exploratory translational studies. Potential correlative analyses include whole-exome sequencing, RNA sequencing, array comparative genomic hybridization, and methylation profiling of biopsy and postprostatectomy specimens. These analyses have not yet been initiated and are not included in the present clinical analysis. If undertaken, the exploratory objective will be to characterize genomic, transcriptional, and epigenetic features potentially associated with biological response to neoadjuvant androgen pathway inhibition and pathological outcomes. Specific laboratory and bioinformatic methods will be defined before initiation of these analyses and reported separately. Surgical and biopsy specimens, as well as serum, urine, plasma, and whole blood, were stored through the CINJ biospecimen bank under deidentification procedures.

### Safety Monitoring

Toxicity monitoring and adverse event reporting were protocol-defined for both apalutamide and abiraterone acetate. Dose modifications were specified for general toxicity, rash, liver function test abnormalities, hypokalemia, hypertension, and edema or fluid retention. Serious adverse events were reported within 24 hours according to institutional and sponsor procedures, and the Human Research Oversight Committee at CINJ served as the data and safety monitoring body for the trial.

Patients in the neoadjuvant arms were monitored prospectively for adverse events using protocol predefined dose holds, reductions, and discontinuation rules, with institutional oversight for safety and compliance.

### Statistical Considerations

The trial was designed around the primary end point of potency recovery by 12 months after RP. On the basis of the principal investigator’s historical surgical outcomes, the surgery-only potency recovery rate was estimated at 13%. Assuming 30 participants per arm, the planned sample size of 90 participants provided 80% power to detect an increase in potency recovery from 13% in the surgery-only arm to 45% in either experimental arm using 1-sided hypothesis testing. Because 2 prespecified experimental-vs-control comparisons were planned (arm 1 vs arm 3 and arm 2 vs arm 3), a Bonferroni-adjusted α level of .025 was applied to each comparison. A sample size of 30 participants per experimental arm was considered sufficient to detect a complete pathological response rate of 20%.

Baseline and study characteristics were summarized using descriptive statistics. Continuous variables were compared using the Wilcoxon rank-sum test, and categorical variables were compared using the Fisher exact test or chi-square test, as appropriate. For the primary end point, arm 1 and arm 2 were each compared separately with the immediate-surgery control arm using 1-sided Fisher exact tests with a prespecified Bonferroni-adjusted significance threshold of α=.025. Unless otherwise specified, additional statistical tests were 2-sided, with *P*<.05 considered statistically significant.

As the study intervention was designed to affect postoperative functional recovery through the surgical procedure, the primary analysis included participants who received the assigned treatment and proceeded through the surgical treatment pathway. To evaluate the robustness of the primary functional analyses to postrandomization withdrawal, a conservative intention-to-treat sensitivity analysis was additionally performed including all randomized participants. Participants who withdrew following randomization were classified as failing the functional end points, not achieving potency recovery at 12 months, and not achieving continence recovery at the assessed postoperative time points.

Biochemical recurrence-free survival was estimated using Kaplan-Meier methods and compared between treatment arms using the log-rank test. Statistical analyses were performed using Stata statistical software (version 18; StataCorp).

## Results

The study was funded by Janssen Scientific Affairs as an investigator-initiated trial. Study accrual began on June 20, 2017, with the final patient accrued in June 2024. A total of 96 patients provided consent and were screened for eligibility, of whom 88 were randomized. Of these, 15 patients did not proceed with their originally assigned treatment following randomization, and 73 patients ultimately received the assigned treatment (arm 1: n=24 [32.8%], arm 2: n=26 [35.6%], and arm 3: n=23 [31.5%]; Figure 2). The study data cutoff occurred on June 30, 2025, following completion of the planned follow-up. A CONSORT (Consolidated Standards of Reporting Trials) diagram summarizing patient flow from screening to inclusion in the analysis is presented in Figure 2. Data analysis is ongoing, and clinical outcomes from this randomized trial will be reported separately from this publication, expected in January 2027.

**Figure 2.**
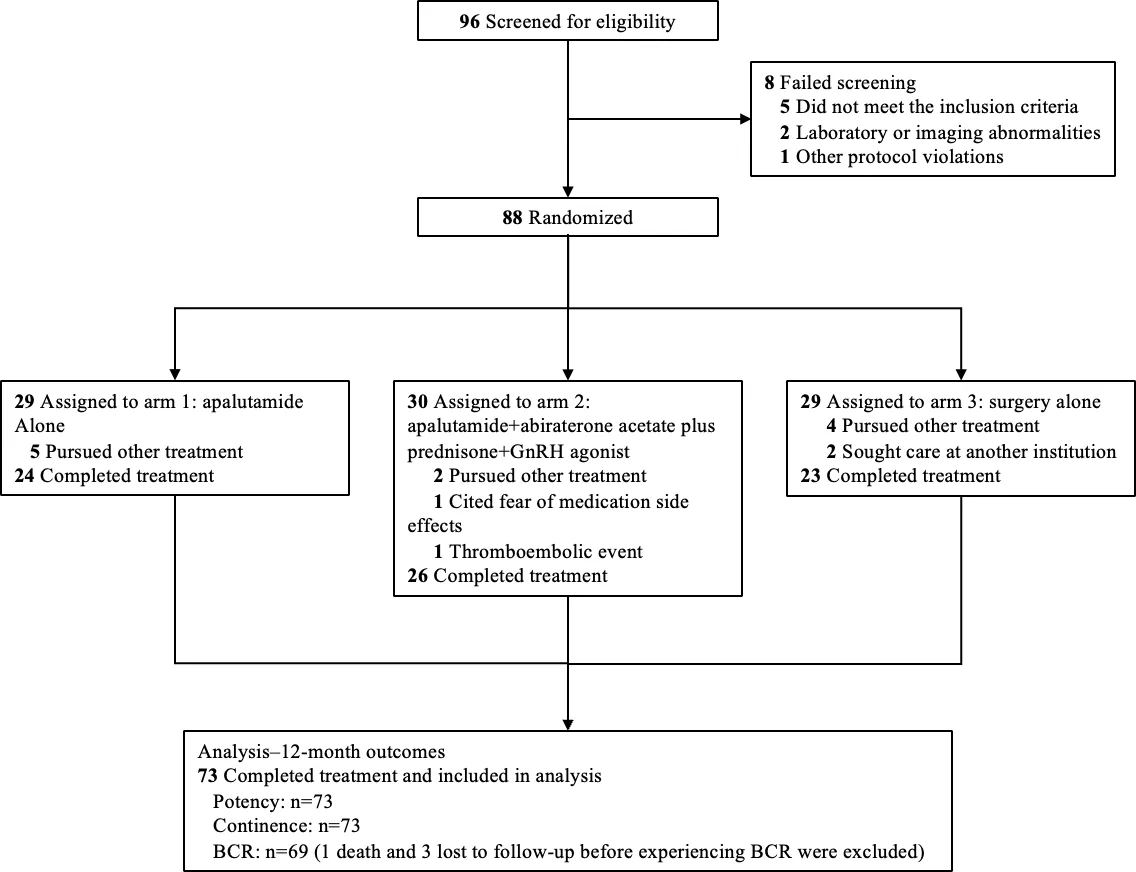
CONSORT (Consolidated Standards of Reporting Trials) flow diagram of patient screening, randomization, and inclusion in the final analysis. A total of 96 patients provided consent and were screened, 88 were randomized, and 73 received assigned treatment. BCR: biochemical recurrence.

## Discussion

This manuscript presents the design and rationale of a randomized phase 2 trial evaluating neoadjuvant apalutamide-based therapy before RP in men with high-risk localized PCa. The study was developed to address a clinically important gap: whether there is a role for neoadjuvant ADT in men with high-risk localized PCa who are undergoing RP.

This protocol is notable for its focus on QoL and functional outcomes. Neoadjuvant ADT is currently not an accepted standard of care because prior studies have focused on oncologic outcomes and shown no significant improvement [12,13]. However, QoL is also an important end point that affects patient satisfaction after cancer surgery. In this context, it is reasonable to use neoadjuvant ADT if QoL improves without adversely affecting oncologic outcomes. Specifically, this trial selected postoperative potency by 12 months as the primary end point and incorporated longitudinal continence recovery data and QoL measures as key secondary outcomes [14,15]. This reflects a clinically meaningful framework, as men considering RP value sexual and urinary function recovery.

A secondary strength of this study is its 3-arm design. By including apalutamide monotherapy, intensified androgen pathway inhibition with apalutamide, AAP, and a GnRH agonist, and immediate surgery, the trial allows comprehensive assessment of whether the addition of intensified androgen suppression may further influence the local tumor environment and functional recovery beyond androgen receptor pathway inhibition alone. This protocol also incorporated serial MRI, prospective toxicity monitoring, and biospecimen collection, which enabled both clinical and translational analyses.

Several limitations inherent to the protocol design should be acknowledged. This is a phase 2 study and was not designed to assess long-term survival benefit. The protocol explicitly states that survival was not an end point because biochemical recurrence typically precedes PCa-specific mortality by many years. Accordingly, the study is best interpreted as a trial of surgical feasibility, functional recovery, and short-term oncologic outcomes. In addition, while the protocol planned to accrue 90 patients (30 per arm), the final treated cohort included fewer patients than originally anticipated for full per-arm completion.

Despite these limitations, this trial addresses an important translational and clinical question in localized high-risk PCa. If neoadjuvant ADT can safely facilitate more favorable surgery and functional recovery, it may broaden the role of preoperative systemic therapy beyond traditional pathological end points. The final study results will aim to clarify whether this approach should be further investigated in larger trials and may inform future neoadjuvant strategies that integrate both oncologic and QoL outcomes.

## Abbreviations

AAP: abiraterone acetate with prednisone
ADT: androgen deprivation therapy
AUA: American Urological Association
CINJ: Rutgers Cancer Institute of New Jersey
CONSORT: Consolidated Standards of Reporting Trials
ECOG: Eastern Cooperative Oncology Group
EPIC: Expanded Prostate Cancer Index Composite
ePLND: extended pelvic lymph node dissection
GnRH: gonadotropin-releasing hormone
MRI: magnetic resonance imaging
NVB: neurovascular bundle
PCa: prostate cancer
PSA: prostate-specific antigen
QoL: quality of life
RP: radical prostatectomy
SHIM: Sexual Health Inventory for Men
